# Differing Water Intake and Hydration Status in Three European Countries—A Day-to-Day Analysis

**DOI:** 10.3390/nu11040773

**Published:** 2019-04-03

**Authors:** Hans Braun, Judith von Andrian-Werburg, Olga Malisova, Adelais Athanasatou, Maria Kapsokefalou, Juan F. Ortega, Ricardo Mora-Rodriguez, Mario Thevis

**Affiliations:** 1 Institute of Biochemistry, German Sport University Cologne, 50933 Cologne, Germany; j.vandrian@dshs-koeln.de (J.v.A.-W.); thevis@dshs-koeln.de (M.T.); 2 German Research Centre of Elite Sports (momentum), German Sport University Cologne, 50933 Cologne, Germany; 3 Unit of Human Nutrition, Department of Food Science and Human Nutrition, Agricultural University of Athens, 11855 Athens, Greece; olgamalisova@yahoo.gr (O.M.); dathanasatou@gmail.com (A.A.); kapsok@aua.gr (M.K.); 4 Exercise Physiology Lab at Toledo, University of Castilla-la Mancha, 13071 Toledo, Spain; juanfernando.ortega@uclm.es (J.F.O.); ricardo.mora@uclm.es (R.M.-R.); 5 Centre for Preventive Doping Research, German Sport University Cologne, 50933 Cologne, Germany

**Keywords:** water, hydration status, total water intake, urine osmolality, urine volume

## Abstract

Adequate hydration is essential for maintaining health and functionality of the human body. Studies assessing both daily water intake and hydration status are lacking. This study explored data from the European Hydration Research Study (EHRS) and focused on total water intake (TWI), 24 h hydration status, and day-to-day variations in a sample of 573 healthy adults. TWI was assessed by food records and hydration status (urine osmolality and urine volume) was measured from 24 urine samples collected over seven consecutive days. On all weekdays, mean TWI was higher (*p* < 0.001 for all days) for the German subjects compared to the Greek and Spanish participants. In 37% of the male and 22% of the female subjects, the individual mean TWI was below the European Food Safety Authority (EFSA) recommendation, with 16% men (4% women) being below the EFSA recommendation on every testing day. Twenty-four hour urine osmolality was lower in women compared to men (595 ± 261 vs. 681 ± 237 mOsmol/kg; *p* < 0.001). More men (40%) showed a urine osmolality ≥800 mOsmol/kg at least on four days of the study period compared to women (26%) and more participants from Spain (46%) compared to Greece (29%) and Germany (11%). A large number of individuals showed an inadequate hydration status on several days per week, which may have a negative health and cognitive impact on daily life.

## 1. Introduction

Water is the main component of the human body and total body water averages about 60% of the body mass in adult males and 50–55% in females [1]. Water is involved in many functions within the human body, such as cellular metabolism, temperature regulation, nerve transmission, cardiovascular transport of oxygen and nutrients [2], as well as the removal of waste products [3]. Optimal physiological function is given in a state of water and electrolyte balance [4,5]. Therefore, adequate hydration and electrolyte homeostasis are essential for maintaining health and functionality of the human body [5].

El-Sharkawy et al. [6] summarized that dehydration has been linked with urological, gastrointestinal, circulatory, and neurological disorders while fluid overload might have an impact on cardiopulmonary disorders, hyponatremia, edema, gastrointestinal dysfunction, and postoperative complications. Studies, which investigated consequences of acute dehydration showed a link between physiological and psychological functions. Predominant alterations concerning physiological sensations that occurred during dehydration were an increased feeling of thirst and dryness of the mouth [7,8], worsened mood [7,9], increasing fatigue [8,10,11,12], and incidences of headaches [7,9,13]. In addition, the short-term memory was affected by dehydration [10,11,14,15], alertness was reduced [8,9,10,11,12,15], and the ability to concentrate decreased [8,9,14,16].

To avoid short and long-term consequences of dehydration, but also overhydration, the European Food Safety Authority (EFSA) recommends a daily total water intake (water from food and beverages) of 2.5 L for men and 2.0 L for women to maintain urinary osmolality of 500 mOsmol/L [1]. Although European Nutrition Surveys show an average fluid intake within this recommended range, low intakes are described for Hungary, Italy, Poland, France, and Slovakia [1,17]. In contrast, mean total water intake is recorded to be well within the EFSA recommendation for Sweden, Netherlands, Germany, Austria, or Ireland. It is unclear if this wide range of water intake data result from different methods used for assessing water intake or if it is based on diet, culture, tradition, availability of drinks, and other factors. However, obviously differences in total water intake between European countries exist. Furthermore, fluid intake and beverage choices vary among individuals in general, but also over the course of a day [17,18,19]. People live, work, and do physical activities in various environments and climatic conditions, which might affect daily water needs. In addition, availability of fluids might differ across a day and therefore hydration status might be suboptimal for some individuals at certain times of a day.

In this context it should be noted that water intake does not automatically describe hydration status. Markers (e.g., serum osmolality, urine osmolality, and urine volume) to assess hydration status in groups or individuals are missing in most dietary surveys [17]. There seems to be a need of studies showing both water intake and hydration status across the European population.

Therefore, the aim of the European Hydration Research Project (EHRS) was to assess water intake and hydration status using a uniform procedure in a sample of healthy adults in three European countries. First results from the EHRS on hydration indices and water intake as well as influence of physical activity and temperature on hydration status have been published recently [20,21]. To our knowledge, data that describe the intra-individual variability in hydration status or total water intake within a selected period are not available. Therefore, this paper will focus on daily total water intake and 24 h hydration status on weekdays with respect to gender and country, but also on intra-individual data on day-to-day variations within the study period.

## 2. Materials and Methods

### 2.1. Study Protocol

This cross-sectional multi-center study was conducted on free-living adults in the metropolitan areas of Cologne (GER), Athens (GRE), and Toledo (SPA) in parallel and following identical protocols during winter (January–March 2013, December 2013, January–February 2014) and summer (June–August 2013, June–July 2014). Subject recruitment was oriented to reach a quota of 25 subjects, balanced for gender (e.g., 12 men and 13 women), in each of the following age groups: 20–30, 31–40, 41–50, and 51–60 years old, in each country. This subject recruitment scheme (100 per country) was repeated in winter and summer with a goal of 200 subjects tested per country (for details please see references [20,21]). Five hundred and seventy three subjects aged 39 ± 12 years (51.1% males) with a BMI 25.5 ± 4.2 kg/m^2^ for males and 24.5 ± 4.9 kg/m^2^ for females were included in the study.

Volunteers were recruited via several access channels (e.g., social media, local newspapers, and local companies). Exclusion criteria were diseases like diabetes insipidus, renal disease, liver disease, gastrointestinal disorders or diseases, cardiac or pulmonary diseases, diseases that limit mobility, and orthopaedic issues. Further exclusion criteria were pregnancy, lactation, hypertensive under severe salt restriction, the intake of drugs that are or contain diuretics, such as phenytoin, lithium, demeclocycline, or amphotericin B, or following a high-protein and/or hypocaloric diet. Demographic factors such as ethnic origin, living conditions, and marital status did not represent exclusion criteria. Subjects were rescheduled or omitted if they had a cold or fever, vomiting, and/or diarrhoea, or if they menstruated during the data collection period. The study protocol was approved by each local Research Ethics Committee (1/26-11-2012 for German Sport University, Germany, 197/27-02-2012 for Agricultural University of Athens, Greece, 4/02/2013-18 for University of Castilla-La Mancha, Spain). Written informed consent was obtained from all subjects.

### 2.2. Study Procedure

The study details were explained in detail to the volunteers during a preliminary talk. All subjects entering the study received a small backpack containing the instruction sheet for the study protocol and material for collecting urine samples and dietary data. For the 24 h urine collection, a diary for recording time of urination and urine volume, a kitchen scale readable to 1 g, a urine collection container, and eight plastic bags containing urine sampling vessels (days 1–7: ten urine sampling vessels, labelled with an individual code, day and number; day 8: one urine sampling vessel for morning urine) were allotted. Furthermore, each subject received a seven-day food record to report in detail time and amount of food and beverages consumed, including wake-up time and bed time. Subjects entered the study on different days of the week in order to achieve a reasonable distribution of starting days over the week. 

On study day 1, subjects arrived fasted at the study center between 7:00–9:30 am bringing a sample of their first morning urine void. Upon arrival, participants’ body height was measured with calibrated mechanical sliding scales and weight was measured with electronic digital scales (±0.05 kg) in underwear and no shoes. Subjects were instructed to sit for approximately 15–20 min while filling in study questionnaires. Subsequently, a blood sample (5 mL) from a forearm vein was collected without stasis. During the study period (days 1–7) subjects were asked to record all food and beverages consumed at the point of intake, following their normal daily routine. The recording was based on measurements with the kitchen scale, or, if that was not possible, portion sizes were estimated based on package information or usual household measures. Participants also collected and recorded the mass of each urination and time of collection and retained a sample in a numbered tube, as instructed. Subjects were asked to store the urine tubes under cool conditions (e.g., refrigerator or in the styrofoam box using fresh ice packs). On day 8, following an overnight fast, subjects visited the laboratory and returned urine samples and the food record; a blood sample was taken and body mass was measured as on day 1. 

Urine collection of each day was from 00:00 to 24:00. A 24 h urine sample was reconstituted from all 24 h recorded and collected urine samples on each day. If subjects reported missing urine samples, 24 h urine was not reconstructed. Urine osmolality was measured with a freezing-point osmometer (GER: Osmomat 3000, Gonotec; GRE: Osmomat 030, Gonotec; SPA: Osmometer 3250, Advanced Instruments Inc.). Urine volume was measured with an electronic digital scale (Soehnle Fiesta 65106) and 1 g of urine rated as 1 mL. Finally, seven-day food records were analyzed for total water intake (TWI) coming from food and beverages using specific softwares and country specific food databases (GER: Ebispro 2011 includes the German Food Database version 3.1, J. Erhardt University of Hohenheim, Stuttgart, Germany; GRE: Diet Analysis plus version 6.1, ESHA Research, Wadsworth Publishing Co. Inc., Salem, OR, USA; SPA: PCN 1.0, CESNID-University of Barcelona, Spain).

### 2.3. Data Processing and Statistical Analysis

Total water intake (TWI) was compared to the EFSA TWI recommendation of 2.5 L for men and 2.0 L for women [1]. Urine osmolality was classified in three groups regarding recommendations to achieve a mean urine osmolality <500 mOsmol/L [1,22], supplemented by suggestions of urine osmolality as a possible sign for hypohydration (>800 mOsmol/L) [22,23]. Finally, daily urine volume was grouped into <1 L/day or ≥1 L/day.

Data entry was performed using Microsoft Excel 2013, Statistical analysis was performed using SPSS (SPSS Statistics 23, IBM, Chicago, IL, USA). Descriptive analysis of variables was conducted indicating data as mean ± standard deviation. Data were tested for normal distribution (Kolmogorov–Smirnov test), plausibility, and consistency. Depending on the existence of normal distribution, parametric tests (T-test or analysis of variance (ANOVA)) or non-parametric tests (Mann–Whitney–U test or Kruskal–Wallis test) were used to analyze group differences. Post hoc comparisons were performed using the Tahame–T2 test. Significance was accepted at the levels 0.001, 0.01, or 0.05, depending on the analysis. The coefficient of variation (CV) is calculated as the ratio of the standard deviation to the mean.

## 3. Results

### 3.1. Total Water Intake

Overall mean TWI of all days was 2.76 ± 1.2 L/day, with a higher TWI intake for men (*p* < 0.001) (Table 1). Mean TWI was similar for most days expect Sundays, which showed a significantly lower TWI (*p* < 0.05) compared to Wednesday, Thursday, Friday and Saturday.

**Figure 1:**
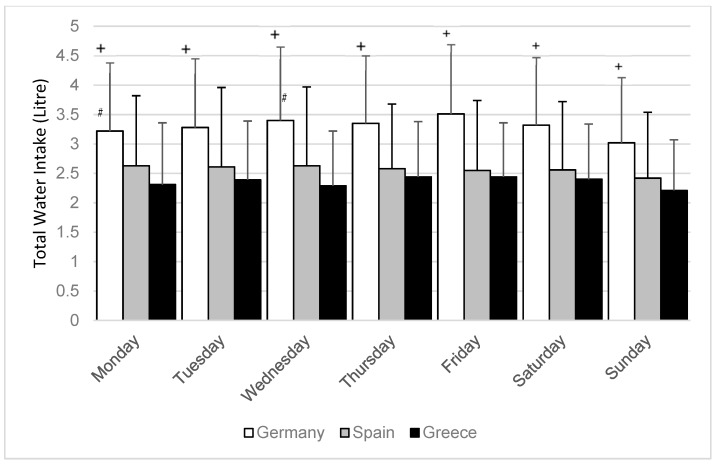
Mean total water intake (L/day) among weekdays with respect to country. *p*-values derived through Tahame–T2 test for comparison between countries. + significantly different on all weekdays for the German subjects compared to the Greek and Spanish (*p* < 0.001). ^#^ significant different for the Spanish subjects compared to the Greek on Mondays and Wednesdays (*p* < 0.05).

TWI was different between countries, with an average TWI of 3.29 ± 0.98 L/day for the German subjects, 2.56 ± 1.01 L/day for the Spanish subjects and 2.34 ± 0.77 L/day for the Greek subjects. With respect to each weekday mean TWI was higher (*p* < 0.001 for all days) for the German subjects compared to the Greek and Spanish participants (Figure 1). On Mondays and Wednesdays TWI intake was higher for the Spanish subjects compared to the Greek (*p* < 0.05).

Mean individual TWI over the seven-day study period below EFSA recommendation was found in 37% males and 22% females. This result varies between countries with a lower rate for Germany (6% males and 7% females) compared to Greece (50% males and 24% females) and Spain (55% males and 39% females). With respect to each single day, 40% of all days from males and 29% from females were <EFSA recommendation (Table 1).

**Figure 2:**
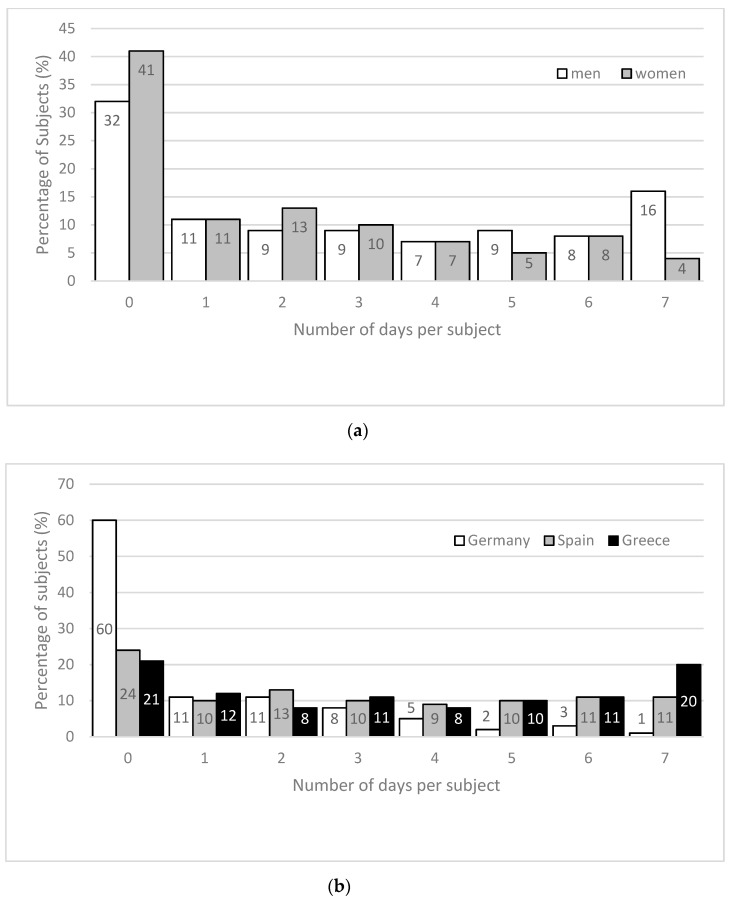
(**a**) Number of days per subject in the seven-day study period with TWI below the EFSA recommendation for men and women; (**b**) number of days per subject in the seven-day study period with TWI below the EFSA recommendation with respect to the country.

Considering the number of days per subjects below EFSA recommendation, 40% of males and 24% of females had at least four days in which TWI was below EFSA recommendation during the seven-day study period (Figure 2a). Furthermore, 16% of all males showed a TWI below 2.5 L/day on every day within the study period. A country-specific analysis demonstrates that 60% of the German subjects (24% of the Spanish and 21% of the Greek subjects) had zero days with a TWI below the EFSA recommendations (Figure 2b). 49% of Greek participants (41% of Spanish and 11% of German participants) showed TWI lower than the EFSA recommendation on at least four days of the study week (Figure 2b).

To describe the intra-individual difference of TWI consumption, the individual highest TWI minus the lowest TWI within the seven-day period was calculated. The overall mean intra-individual difference was found to be 1.67 ± 0.94 L. The coefficient of variation (CV) for TWI was 20.5% ± 9.2%.

### 3.2. Urine Osmolality

On each weekday, 24 h urine osmolality is lower (*p* < 0.01 for each day) in females compared to males (Table 2).

We observed more 24 h urine samples with an osmolality <500 mOsmol/kg in women (44%) compared to men (26%). Twenty-four hour urine samples with an osmolality ≥800 mOsmol/kg occurred more often in males than in females (32% vs. 24%). This trend exists on all weekdays (Table 3).

**Figure 3:**
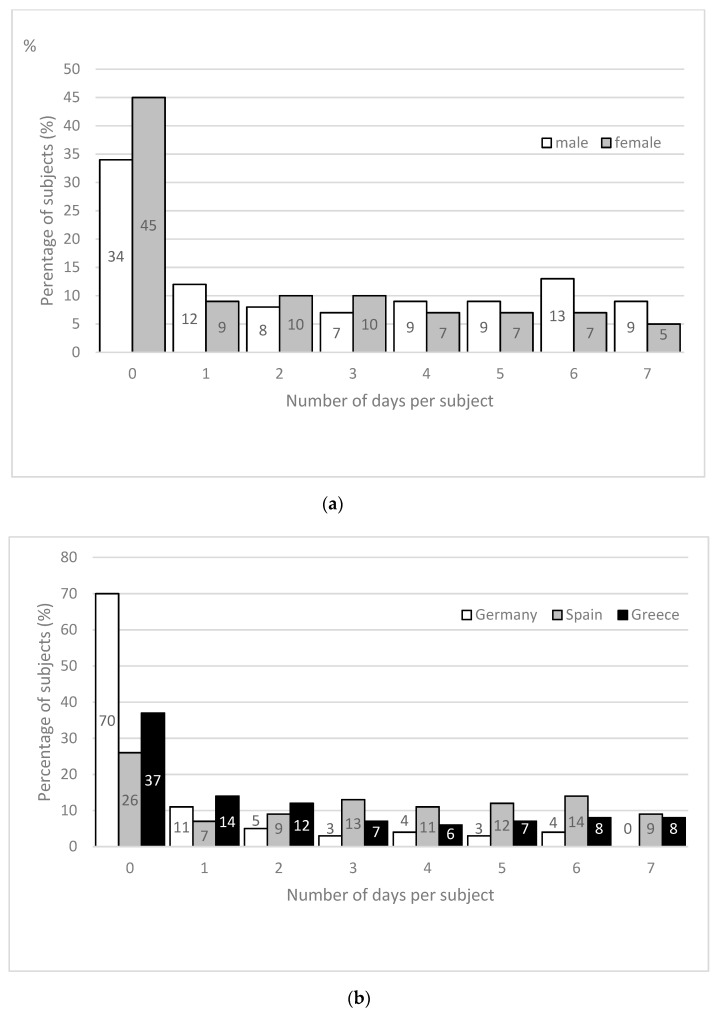
(**a**) Number of days per subject in the seven-day study period with a 24 h urine osmolality >800 mOsmol/kg for men and women; (**b**) number of days per subject in the seven-day study period with a 24 h urine osmolality >800 mOsmol/kg with respect to the country.

Overall, 11% of the study population (6% men; 15% women) showed a 24 h urine osmolality on all days of the study period <500 mOsmol/kg. In contrast, 9% of all men (5% women) showed a urine osmolality ≥800 mOsmol/kg on all seven days. Almost half (40%) of the male participants and 26% of the female participants had a urine osmolality ≥800 mOsmol/kg on at least four days within the study week (Figure 3a).

A country specific analysis illustrates that 70% of the German subjects (26% SPA, 37% GRE) had zero days with urine osmolality ≥800 mOsmol/kg during the study period. In contrast, 46% of the Spanish participants (29% GRE, 11% GER) showed a urine osmolality ≥800 mOsmol/kg on at least four days of the study week (Figure 3b) including almost 10% of the subjects in Greece and Spain with a 24 h urine osmolality ≥800 mOsmol/kg on every day of the study period. The CV for urine osmolality was 17.9% ± 8.6%.

### 3.3. Urine Volume

Mean daily urine volume was found to be 1.68 ± 0.85 L/day with no difference between weekdays and gender. However, 19% of the men and 24% of the women had a urine volume <1 L/day (Table 4). The CV for urine volume was 23.2% ± 11.2%.

## 4. Discussion

The European Hydration Research Study (EHRS) is the first study to examine total water intake and selected hydration markers over a period of seven consecutive days in healthy adults. While studies in this field are quite often not comparable due to different collecting methods and survey dates [18], we conducted the study following a normalized experimental protocol and methods in all three countries. In this study, we found differences in TWI and 24 h urine osmolality with respect to country and gender. While mean group results are within the recommended levels, individual data show an intra-individual variation in daily TWI and 24 h urine osmolality.

### 4.1. Total Water Intake

Overall mean TWI of the study population is in the range of the suggested EFSA adequate intake [1] for both men and women. The country-specific difference in our study is comparable to the variance in TWI among European countries described by others already [1,17,19]. Individual mean TWI of the seven-day study period was below the EFSA recommendation for 37% men and 22% women. This overall result is analogous to data from the UK [19]. Sunday was found to be the day with the lowest TWI in all countries (Figure 1); this should not be misinterpreted as particularly low, as TWI on Sunday is only about 200 mL below the overall mean TWI intake. However, while this observation is similar for females from a UK population, it is different from UK males who reported lowest beverage consumption on Monday [19].

Deeper analysis of the daily data reveals that a substantial amount of all subjects (men 40%; women 24%) had TWI below EFSA on ≥4 days and almost every fourth male below EFSA on ≥6 days per week (Figure 2a). These results emphasize that although the overall mean TWI is within the recommended values, an intra-individual variance regarding TWI within a seven-day period exists. Such a day-to-day difference in beverage consumption habits is already described [18] and supported by our findings.

Based on previous results, the German subjects showed a higher water intake from beverages and a higher water intake from food [20]. However, at this stage, we are unable to explain why we found more men compared to women and more people from Greece and Spain being unable to meet the EFSA recommendation compared to the German subjects. While misreporting/underreporting in dietary surveys is common [1], the extent of beverage misreporting is not known for the sedentary population [18], and to our knowledge country-specific underreporting has not been described so far. However, the day-to-day variety of TWI might have implications on short-term surveys (e.g., 24 h recalls) and should be kept in mind when planning surveys on TWI and hydration status [18].

In addition, it remains unclear what consequences the day-to-day variety has in each individual of our study. Water requirements and TWI vary highly between individuals with respect to daily activities, diet, climate, and environment. Therefore, based on TWI data solely, it is difficult to determine how many subjects were hypohydrated during the study period or on selected days.

### 4.2. Urine Osmolality

Although no single biomarker represents hydration status in humans in all situations and persons, 24 h urine osmolality is regarded as an excellent indicator, as it represents the sum of all behavioral and neuroendocrine responses and the whole body hydration status more accurately than single spot samples [23,24,25]. Urine osmolality varies within 50 and 1200 mOsmol/L with a theoretical maximum of 1400 mOsmol/L [1]. However, a maximum urine osmolality in adults has been determined to be 900–1400 [1]. A 24 h urine osmolality <500 mOsmol/kg is suggested to be desirable to excrete the daily solute load [1,22] and urine osomolality >800 mOsmol/kg is suggested to represent mild hypohydration [22,23,26,27].

Individual water requirement is based on respiratory and sweat losses but is also dependent on the diet and its osmotic solute content and the concentrating capacity of the kidneys [1]. For safety concerns regarding kidney health, daily water intake recommendation is connected with a urine osmolality of about 500 mOsmol/kg [1,22]. In this study the female subjects showed a significantly lower 24 h urine osmolality compared to males. However, the mean values were above the suggested 500 mOsmol/kg for both males and females on each weekday. With respect to the EFSA recommendation to achieve a daily 24 h urine osmolality <500 mOsmol/kg [1], we found only 11% of our subjects reached this value on a daily basis. This appears alarming following the suggestion that a urine osmolality <500 mOsmol/kg is a relevant physiological index of hydration for the general population [22]. Furthermore, in 40% of all males we observed a 24 h urine osmolality ≥800 mOsmol/kg on ≥4 days in the seven-day study period with enormous difference between the three countries. This can be interpreted as a large number of days in which our subjects might have had inadequate water consumption [23]. Even though we don’t know if our results describe a long-term behaviour and what consequences 24 h urine osmolality ≥800 mOsmol/kg has on each individual, an increased TWI for those subjects seems to be necessary. Attention should be paid to specific groups to reduce possible risks on chronic kidney disease [28,29,30,31], although well-designed prospective studies are needed before such a recommendation can be justified [32].

Assuming this describes a typical behavior of our subjects, detrimental effects on wellbeing, mood, or health are possible [6,33]. Mild hypohydration can cause symptoms like dizziness, headache, or fatigue with lower self-reported ratings of alertness and ability to concentrate [7,8,9,10,11,12,13,14,15,16,33]. Depending on the work to be done on such days, this possibly has a negative impact on several situations during daily life. Recently, an increased number of driving errors during a prolonged, monotonous drive were reported when subjects were hypohydrated [34].

### 4.3. Urine Volume

Using urine volume as a marker for hydration status, it should be kept in mind that physical activity and heat decrease urine output, while cold and hypoxia increase it [1]. Urine volume, TWI, and urine osmolality are closely related [1,24], and urine volume varies inversely with the body hydration status [35]. Average urine volumes in adults are described to be 1–2 L/day with extremes in both directions [1,35]. An average urine output of approximately 100 mL/h in healthy people is possibly a sign of being well hydrated. In contrast, if urine output decreases to an average of 30 mL/h the person is probably dehydrated [35]. However, an agreement on a urine volume to describe hypohydration does not exist. Suggestions exist that the minimum volume that must be excreted generally amounts to 20 to 50 mL/h [36], which results in a basal urine volume in the range of 480 to 1200 mL/day. Within our study we found a mean urine volume on all weekdays for males and females within the described values. However, 24% of the female 24 h urine samples and 19% of the male were below 1 L/day. While we found no gender specific difference on urine volume, previous results from the EHRS project showed that urine volume of the German subjects was significantly higher compared to the Greek and Spanish participants [20]. This is likely due to the higher TWI intake, which also explains the lower urine osmolality of the German subjects [20,21].

## 5. Conclusions

In Summary, we highlighted TWI, 24 h urine osmolality, and urine volume in a group of 573 adults from three different European countries. We found differences in TWI and 24 h urine osmolality between countries and between males and females. Mean group results from a seven-day data collection are within the recommended levels. However, individual data show an intra-individual day-to-day variation in TWI and urine osmolality. Individuals were identified with low TWI and high 24 h urine osmolality on several days per week or even daily. While it is unclear what the consequences are for the individual subject currently tested, chronic hypohydration may have detrimental effects on wellbeing, mood, or health [7,8,9,10,11,12,13,14,15,16,33,34]. Future studies need to consider the intra-individual day-to-day variation and show if this might have consequences on health and wellbeing.

## Figures and Tables

**Table 1:** Mean total water intake (TWI) on all weekdays and percentage of subjects with TWI below European Food Safety Authority (EFSA) recommendation.

Weekday	Total Water Intake (Mean ± SD in L/day)				TWI < EFSA (in %)		
	All	Men	Women	*p*-Value *	All	Men ^#^	Women ^#^
Monday	2.74 ± 1.2	2.92 ± 1.26	2.55 ± 1.08	=0.001	35	41	30
Tuesday	2.78 ± 1.2	2.94 ± 1.41	2.62 ± 1.04	=0.012	32	38	25
Wednesday	2.80 ± 1.3	3.00 ± 1.38	2.59 ± 1.13	<0.001	35	39	30
Thursday	2.80 ± 1.1	2.97 ± 1.21	2.64 ± 1.03	=0.002	32	38	25
Friday	2.85 ± 1.2	3.02 ± 1.32	2.69 ± 1.08	=0.004	35	41	28
Saturday	2.78 ± 1.2	2.98 ± 1.29	2.59 ± 0.99	=0.001	35	41	28
Sunday	2.57 ± 1.1	2.76 ± 1.19	2.38 ± 0.98	<0.001	37	41	36
All days	2.76 ± 1.2	2.94 ± 1.10	2.57 ± 0.89	<0.001	35	40	29

* *p*-values derived through Student’s *t*-test for differences between genders; ^#^ EFSA recommendation for men 2.5 L/day and women 2.0 L/day. TWI: Total water intake; EFSA: European Food Safety Authority recommendation.

**Table 2:** Mean 24 h urine osmolality (mOsmol/kg) on weekdays for men and women.

Weekday	24 h Urine Osmolality (Mean ± SD in mOsmol/kg)			
	All	Men	Women	*p*-Value *
Monday	642 ± 252	680 ± 238	605 ± 259	<0.001
Tuesday	639 ± 256	687 ± 238	593 ± 265	<0.001
Wednesday	620 ± 255	670 ± 242	568 ± 257	<0.001
Thursday	639 ± 265	685 ± 250	590 ± 270	<0.001
Friday	634 ± 254	669 ± 234	595 ± 267	<0.001
Saturday	637 ± 250	686 ± 239	588 ± 252	<0.001
Sunday	656 ± 241	687 ± 216	624 ± 260	=0.002
All 24 h samples	638 ± 254	681 ± 237	595 ± 261	<0.001

* *p*-values derived through Mann–Whitney–U test for differences between genders.

**Table 3:** Percentage of 24 h urine osmolality within selected categories on weekdays for men and women.

Weekday	<500 mOsmol/kg (%)			500–799 mOsmol/kg (%)			≥800 mOsmol/kg (%)		
	All	Men	Women	All	Men	Women	All	Men	Women
Monday	34	27	41	36	39	33	30	34	26
Tuesday	33	23	43	38	42	34	29	35	23
Wednesday	38	29	47	36	39	33	26	32	20
Thursday	35	25	45	36	41	32	29	34	23
Friday	36	28	46	35	40	30	28	32	24
Saturday	35	26	45	39	43	34	26	31	21
Sunday	30	22	39	40	47	32	30	31	29
All 24 h samples	34	26	44	37	42	33	28	32	24

**Table 4:** Mean 24 h urine volume (L/day) on weekdays and percentage of subjects with a urine volume <1 L/day, for men and women.

Weekday	24 h Urine Volume (Mean ± SD in L/day)				Urine Volume <1 L/day (%)		
	All	Men	Women	*p*-Value *	All	Men	Women
Monday	1.69 ± 0.87	1.69 ± 0.83	1.69 ± 0.91	=0.615	21	18	24
Tuesday	1.66 ± 0.86	1.64 ± 0.80	1.69 ± 0.91	=0.907	24	24	24
Wednesday	1.70 ± 0.89	1.68 ± 0.86	1.73 ± 0.91	=0.683	22	22	22
Thursday	1.66 ± 0.80	1.68 ± 0.78	1.66 ± 0.83	=0.418	22	17	26
Friday	1.70 ± 0.88	1.69 ± 0.81	1.73 ± 0.95	=0.785	20	17	23
Saturday	1.68 ± 0.85	1.65 ± 0.79	1.71 ± 0.92	=0.911	21	19	23
Sunday	1.65 ± 0.82	1.68 ± 0.79	1.63 ± 0.85	=0.198	22	17	26
All 24 h samples	1.68 ± 0.85	1.67 ± 0.81	1.69 ± 0.90	=0.881	22	19	24

* *p*-values derived through Mann–Whitney–U test for differences between genders.
